# Prospects of biotechnological production and adoption of COVID-19 serological assays in Nigeria

**DOI:** 10.11604/pamj.supp.2020.37.1.25823

**Published:** 2020-11-03

**Authors:** Idris Nasir Abdullahi, Kabir Umar, Pius Omoruyi Omosigho

**Affiliations:** 1 Department of Medical Laboratory Science, Faculty of Allied Health Sciences, Ahmadu Bello University, Zaria, Nigeria,; 2 Department of Medical Microbiology and Parasitology, Bayero University, Kano, Nigeria,; 3 Department of Medical Laboratory Science, Kwara State University, Mellete, Nigeria

**Keywords:** COVID-19 serology, laboratory tests, biotechnology, Nigeria

## To the Editor of the Pan African Medical Journal

For over 8 months of its emergence, the Severe Acute Respiratory Syndrome Coronavirus 2 (SARS-CoV-2), the causative agent of the Coronavirus Disease 2019 (COVID-19) pandemic has resulted to unprecedented global health challenge and economic uncertainties. Till date, the World Health Organization (WHO) recommended the Reverse Transcription-Polymerase Chain Reaction (RT-PCR) as the gold standard for COVID-19 diagnosis. As at 30^th^ August 2020, there were 53727 RT-PCR confirmed COVID-19 cases out of the 398304 tested persons in Nigeria [1]. Even though the government of Nigeria has been activating some laboratories to scale-up its testing capacity, many observers have attributed the relatively low reported COVID-19 cases to underdiagnosis, probably due to inadequate molecular diagnostic capacity and few human resources skilled in molecular diagnostic tests [2]. Although appropriate use of RT-PCR provides very accurate results, test reagents and consumables are mostly in short supply. Besides, this protocol is laborious, expensive to operate and has a long test turnaround time (TAT) [3]. Aside from these, one of the major technical drawbacks in the use of RT-PCR includes significant cases of false-negative results despite patients having clinical features and radiologic findings highly suspicious of SARS-CoV-2 infection [4]. This could have been due to wrong sampling where SARS-CoV-2 might have been present in the lower respiratory tracts rather than upper respiratory samples often collected for laboratory diagnosis. This poses a challenge in the proper evaluation of some SARS-CoV-2 infected persons.

It has been observed that the transmission dynamics of SARS-CoV-2 has made it a herculean task in its control in Nigeria. Particularly, with the recent identification of a mutant (D614G), a highly transmissible form of SARS-CoV-2 in Nigeria. This necessitates the need to “think out of the box” by searching for highly sensitive and specific anti-SARS-CoV-2 protocols with short TAT. Thus, enabling prompt and large-scale testing for COVID-19. Even though none of the antibody-based Point-of-Care-Test (POCT) devices have been approved by the WHO, a number of antigen (Ag)-based serological assays have been approved for use. Antigen-based serological tests directly detect SARS-CoV-2 proteins of replicating virus in respiratory secretions. This assay has been developed as both ELISA, and for POCT. Most Ag-based POCTs for SARS-COV-2 use the sandwich immunodetection protocol that employs a simple-to-use lateral flow assay (LFA) format commonly employed for other common infectious diseases testing [5]. Available performance characteristics for Ag-based POCTs were derived from studies of different designs. So far, Ag-based POCTs on upper respiratory tract samples have shown variable sensitivity when compared to NAATs, ranging from 0-94%. However, their specificities were consistently reported to be high (>97%) [5]. Although there is the need for more evidence on real-world performance and operational issues, Ag-based POCTs will most likely perform well in patients with high viral loads which usually appear in from day 1 to 3 before the onset of symptoms (pre-symptomatic) and during the early symptomatic periods of COVID-19 (usually from 5-7 days of illness). This could offer an opportunity for prompt diagnosis and interruption of transmission through targeted isolation of most infectious cases and their close contacts [5]. Despite these expected limitations in performance, if correctly performed and interpreted, Ag-based COVID-19 POCTs could have a significant application in patient management, surveillance and other public health decision making [5]. Diagnostically, Ag-based POCTs need to have sensitivity ≥80% and have very high specificity of ≥97-100% [5]. Furthermore, several reports have demonstrated the ability of antibody-based POCTs to assist in the sero-epidemiological analyses of the ongoing COVID-19 pandemic. The immuno-kinetics of SARS-CoV-2 infection shows that serum anti-SARS-CoV-2 IgM is detectable by the third day after infection, while anti-SARS-CoV-2 IgG levels rise when anti-SARS-CoV-2 IgM levels begin to decline at day 7 [6]. Essentially, the technical rigor and quality of specimen requirements for these tests are relatively lesser than those of RT-PCR protocols.

So far, two major concerns with serological tests are the possibility of missing acute SARS-CoV-2 infection during “window period” and the possibility to “cross-reaction” with some SARS-CoVs, due to their high nucleotide homology with SARS-CoV-2 [7]. Despite these, the performance characteristics of various serological assays were incredibly encouraging. For instance, the Cellex® qSARS-CoV-2 IgM/IgG POCT devices showed 96.7%, 96%, 92.3% and 96.8% sensitivity, specificity, positive predictive and negative predictive values, respectively [8]. To attain sufficient public health laboratory response required for the adequate containment of the COVID-19 pandemic in Nigeria, the National Biotechnology Development Agency; National In-vitro Diagnostics Control Laboratory; private biotechnology firms and regulatory authorities should consider local production, post-production evaluation and standardization of POCT devices to scale-up Nigeria´s COVID-19 testing capacity viz a viz avoid inaccurate results. By this, many biomedical science-related professionals will be employed. Besides, Nigeria could consider the exportation of quality assured COVID-19 POCT devices to neighboring African countries. This will ameliorate the impact of the COVID-19 pandemic on Nigeria´s health economy.
